# The mechanisms and mechanical energy of human gait initiation from the lower-limb joint level perspective

**DOI:** 10.1038/s41598-021-01694-5

**Published:** 2021-11-18

**Authors:** Guoping Zhao, Martin Grimmer, Andre Seyfarth

**Affiliations:** Lauflabor Locomotion Laboratory, Centre for Cognitive Science, Technical University of Darmstadt, 64289 Darmstadt, Germany

**Keywords:** Biomedical engineering, Musculoskeletal system, Motor control, Scientific data

## Abstract

This study aims to improve our understanding of gait initiation mechanisms and the lower-limb joint mechanical energy contributions. Healthy subjects were instructed to initiate gait on an instrumented track to reach three self-selected target velocities: slow, normal and fast. Lower-limb joint kinematics and kinetics of the first five strides were analyzed. The results show that the initial lateral weight shift is achieved by hip abduction torque on the lifting leg (leading limb). Before the take-off of the leading limb, the forward body movement is initiated by decreasing ankle plantarflexion torque, which results in an inverted pendulum-like passive forward fall. The hip flexion/extension joint has the greatest positive mechanical energy output in the first stride of the leading limb, while the ankle joint contributes the most positive mechanical energy in the first stride of the trailing limb (stance leg). Our results indicate a strong correlation between control of the frontal plane and the sagittal plane joints during gait initiation. The identified mechanisms and the related data can be used as a guideline for improving gait initiation with wearable robots such as exoskeletons and prostheses.

## Introduction

Adults typically walk between 6000 and 13,000 steps per day^1^. In addition to longer episodes of walking, there are many short episodes where transitions between the stable state of standing and the dynamically stable state during walking are required. Aside from keeping balance during transitions, gait termination must dissipate energy while gait initiation must inject energy to the body segments.

Gait initiation could be challenging for human balance control system as it transitions from static standing balance to dynamic periodic walking balance^2^. For instance, patients with Parkinson’s disease often have difficulties in initializing walking from standing^3,4^. From the lower-limb muscle activation perspective, gait initiation starts with an abrupt decrease in the soleus and gastrocnemius muscle activation with an associated increase in the tibialis muscle activation on the stance limb^5–8^.

Several studies have also investigated the center of mass (CoM) and center of pressure (CoP) movements during gait initiation. The gait initiation process can be described as following: To start gait initiation, the CoP rapidly moves posteriorly and towards the swing limb to accelerate the CoM forward and towards the stance limb^7,9–11^. Then, the swing limb is getting unloaded and the CoP moves towards the stance foot, creating an acceleration forward^7,9–11^. Before touch down of the swing foot, the CoM has already established a near steady-state trajectory^10^. However, it remains unclear how lower-limb joints contribute to the mechanism of shifting the CoM and CoP.

Based on inverse dynamics, lower-limb joint mechanical power and energy have been used to explain walking energetics^12,13^. Positive and negative mechanical joint power indicate energy injection and energy dissipation/storing, respectively. For level walking the average positive and negative power of the hip, knee and ankle increase as walking velocity increases^13^. Similarly, joint work and peak power increase^14,15^. In contrast, the relative contribution of total average positive power for each lower-limb joint remains at an equal level^13^. For instance, the hip and the ankle provide the major energy injection (about 40%) during walking at velocities of 0.75–2.0 m/s^13^. For gait initiation, Hansen et al.^16^ analyzed ankle energy contribution and demonstrated that the ankle net positive work increases with increasing gait initiation target velocity. To date, no study has shown the lower-limb joint mechanical power and energy contribution during gait initiation of different target velocities.

This study aims to improve our understanding of the energy sources and processes that are used during gait initiation to create and increase the forward velocity of the CoM. The analysis looks at the local joint level as well as the global CoM behavior. We expect that humans use a combination of specific energy injection strategies to achieve a target velocity, including (a) a dominant role of specific joints to inject energy, (b) transfer from potential to kinetic energy, and (c) avoiding of energy dissipation. Further, we expect that there are (d) actively controlled weight shifting balance mechanisms that are coordinated between the frontal and the sagittal planes in order to organize the energy injection.

In order to study the target velocity related effects on the joint contributions during gait initiation, three different target velocities were analyzed in our study. Farris and Sawicki^13^ found that different walking velocities have a similar relative contribution of total average hip, knee and ankle positive power. We expect that, in line with the previous findings^13^, relative energetic joint contributions and the balance mechanisms do not change with target velocity. We explain our expectations in more detail below.

First, in continuous level walking the hip and the ankle joint contribute a similar amount of positive work while the knee contributes less than half of what the hip or the ankle do^13^. We want to investigate if this relationship also exists during gait initiation. More specifically, we focus on investigating if there are specific joints that primarily drive the leading and trailing limbs in the initiation stride and in the following strides to reach the target velocity. It is hypothesized that, in order to swing the leg forward, the hip in the leading limb contributes more during the first initiation stride compared to steady state walking. For the following strides, we expect a relative contribution of all joints similar to steady state walking with major positive work contributions at the hip and ankle.

Second, we expect there are two major energy sources that contribute to the increase of the CoM kinetic energy: (1) lower-limb joint positive work, and (2) CoM potential energy. We hypothesized that the potential energy primarily contributes to the CoM kinetic energy for the first stride, whereas the positive joint work dominates in the acceleration strides that follow.

Third, in addition to energy injection during gait initiation, reducing energy dissipation could be an energy efficient strategy for acceleration. It is hypothesized that the CoM mechanical collision work and preload work are reduced compared to regular walking, but these quantities increase with greater walking velocity.

Finally, at the beginning of gait initiation, the CoM initially shifts towards the trailing limb^10,17^. To investigate the mechanism of this shift, we focus on joints that contribute to lateral sway. There are two main mechanisms that could initiate the lateral shifting of the CoM: (1) hip abduction torque on the leading limb side and/or hip adduction torque on the trailing limb side, and (2) elongating the leading limb (e.g. ankle dorsiflexion) and/or shortening the trailing limb (e.g. knee flexion). As elongating or shortening the leg might conflict with the initiation of sagittal plane movement, we expect that it is easier to achieve the CoM shift with hip abduction or adduction. We hypothesized that this is primarily accomplished by hip abduction on the leading limb because the hip also exerts large abduction torque during the stance phase of regular walking^18^.

## Results

The results section consists of two subsections. The first subsection focuses on the mechanism of the gait initiation based on the joint level and the CoM data. The second subsection focuses on the energy injection from the average CoM power and each lower-limb joint.

In the following figures, the results are presented with respect to the first five strides during the gait initiation. L1, L2 and L3 denote the first, the second and the third stride of the left leg, respectively. R1 and R2 denote the first and the second stride of the right leg. Ref denotes the reference stride which is steady overground walking at self-selected preferred velocity. The results in the Ref condition are calculated with both left and right sides across all subjects.

### Gait initiation mechanism

We separate the gait initiation process into the following four different phases: the weight shifting initiation phase (P1), the leading limb lifting initiation phase (P2), the trailing limb push-off phase (P3), and the stabilizing phase (P4).

#### Weight shifting initiation phase (P1)

**Figure 1:**
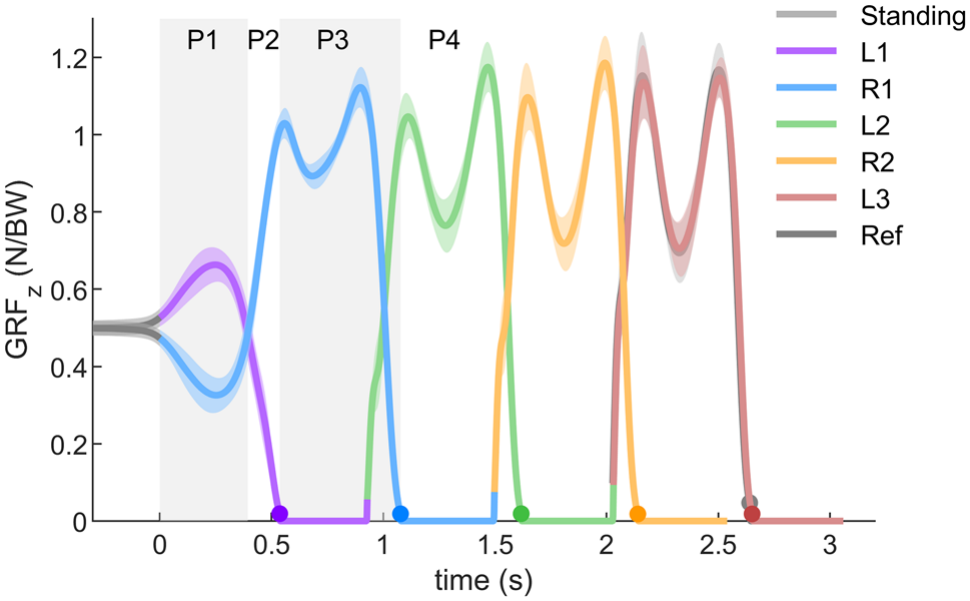
Vertical GRF ($\text {GRF}_\text {z}$) during normal velocity gait initiation. $\text {GRF}_\text {z}$ is normalized to subject body weight (BW). The solid lines denote the average $\text {GRF}_\text {z}$ over 23 subjects. The error bands denote the $\pm 1$ standard deviation. Time 0 is defined as the start of gait initiation. The dots denote the take-off (TO) moment. P1: weight shifting initiation phase; P2: leading limb lifting initiation phase; P3: trailing limb push-off phase; P4: stabilizing phase.

**Figure 2:**
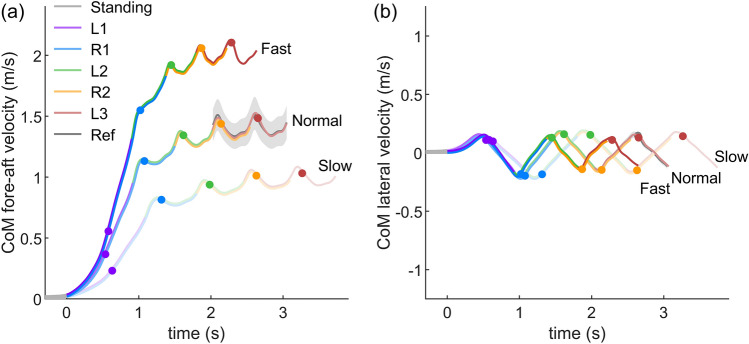
Mean CoM velocity over 23 subjects for the slow, normal and fast target velocity gait initiation. Ref denotes the regular overground walking at the self-selected preferred velocity. The light gray error band indicates the $\pm \,1$ standard deviation of Ref. The error band of each stride is not shown to keep the figure easy to read. Time 0 is defined as the start of gait initiation. Different colors denote different strides. The dots denote the take-off timing. (**a**) The CoM velocity in the fore-aft direction. Positive values indicate the forward direction. (**b**) The CoM velocity in the lateral direction. Positive values implies the CoM is moving towards the subject’s right-hand side (the trailing limb side).

**Figure 3:**
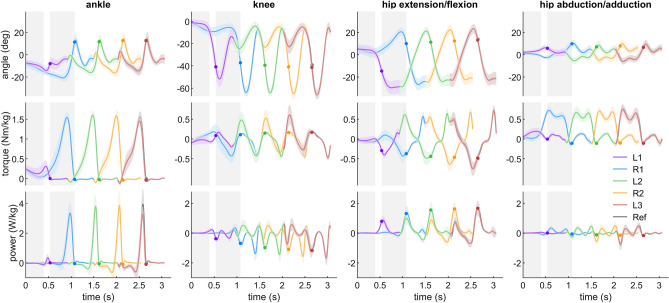
Ankle extension/flexion, knee extension/flexion, hip extension/flexion and hip abduction/adduction angle, torque and power during normal velocity gait initiation. Hip and knee extension, ankle plantarflexion, hip abduction, and extension torque are defined as positive. Solid lines denote the mean value over 23 subjects. The error bands denote $\pm \,1$ standard deviation. Time 0 is defined as the beginning of the gait initiation. Different colors denote different strides. L1, L2, L3 denote the first, second, and third stride on the left side, respectively. R1 and R2 denote the first and second stride on the right side, respectively. Ref denotes the regular overground walking at the self-selected preferred velocity. The dots denote the take-off timing.

The weight shifting initiation phase is defined from the beginning of gait initiation (see “Methods” section) to the beginning of hip flexion, which occurs with the instance of equal vertical ground reaction force values of L1 and R1 (Fig. 1). During the weight shifting phase, the CoM shifts forward and laterally towards the trailing limb (Fig. 2). To realize the lateral shift, the hip of the leading limb (L1) introduces an abduction torque (Fig. 3). With this increasing hip torque, we observe an instantaneous increase in the vertical GRF of the leading limb and a decrease in the vertical GRF of the trailing limb (L1 and R1 in Fig. 1). The timing of the vertical GRF peak of L1 and R1 (negative), and the peak of the L1 hip abduction torque are aligned. At the trailing limb, the vertical GRF decreases while the hip, knee and ankle joints are flexing (Figs. 1, 3). While the ankle extension torques on both limbs are decreasing (Fig. 3), a forward shifting of weight is observed (Fig. 2).

#### Leading limb lifting initiation phase (P2)

The initiation of lifting the leading limb starts with hip flexion and ends with the take-off of the leading limb. Simultaneously, the hip flexion torque and the hip flexion angle increase (Fig. 3). Small amounts of positive power in ankle plantarflexion and knee flexion can be observed (Fig. 3). While the hip angle is much more flexed compared to the reference walking stride, the magnitude of the flexion torque, and particularly the flexion power are comparable (Fig. 3).

#### Trailing limb push-off phase (P3)

The trailing limb push-off phase starts after the take-off of the leading limb and ends with the take-off of the trailing limb. It is characterized by high ankle plantarflexion and knee flexion torque as well as increasing values in hip flexion torque (Fig. 3). A high burst of positive ankle plantarflexion power, positive hip flexion power and small amounts of knee positive power can be observed during this phase. At the first touch-down of the leading limb (the beginning of L2), the knee and hip flexion angles are larger than the reference walking trial (Fig. 3).

#### Stabilizing phase (P4)

The stabilizing phase starts at the push-off of the trailing limb. It is characterized by similar joint angle, torque and power patterns compared to the reference walking stride (Fig. 3). The amplitudes of the reference walking stride are reached with increasing walking velocity. The majority of the target velocity is already achieved at take-off of the trailing limb (R1) and only small increases are found for L2, R2 and L3 for all tested target velocities (Fig. 2, Supplementary Table S1).

### Energy injection during the gait initiation

#### Joint energy input

**Figure 4:**
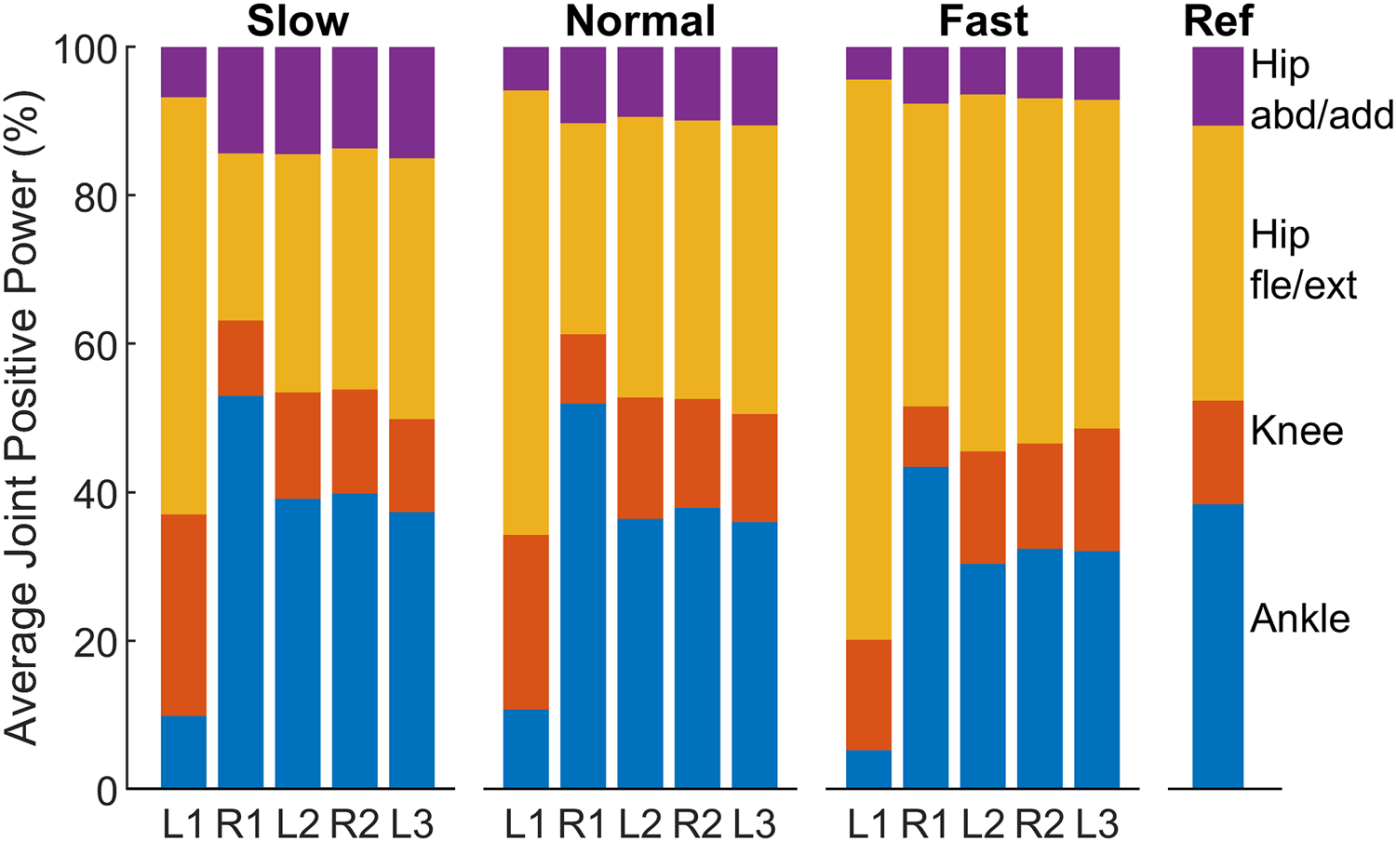
Relative contribution of the average positive joint power for the first five strides of slow, normal and fast gait initiation and for the reference trials (Ref). Purple indicates the hip abduction/adduction joint. Yellow indicates the hip flexion/extension joint. Orange indicates the knee flexion/extension. Blue indicates the ankle plantarflexion and dorsiflexion.

For the first stride of the leading limb (L1), more than half of the average positive joint power is provided by the hip flexion/extension joint (slow $56.2\%\pm 14.2$%, normal $60.0\%\pm 14.9$%, fast $75.5\%\pm 10.8$%, Fig. 4), which is higher than the reference stride (steady walking at preferred velocity, $37.1\%\pm 6.2$%). During L1, the ankle joint provides very little contribution (slow $9.8\%\pm 9.7\%$, normal $10.7\%\pm 8.4\%$, fast $5.2\%\pm 4.4\%$), which is much lower than the reference stride ($38.4\%\pm 5.2\%$). In contrast, for the first stride of the trailing limb (R1), the ankle joint contributes approximately half of the total average positive power in slow ($52.9\%\pm 6.7\%$) and normal ($51.9\%\pm 5.7\%$) target velocity condition, which is greater than the reference stride ($38.4\%\pm 5.2\%$). The relative joint contribution for providing positive joint power changes with each stride until L2 and it stays almost equal in the following strides.

**Figure 5:**
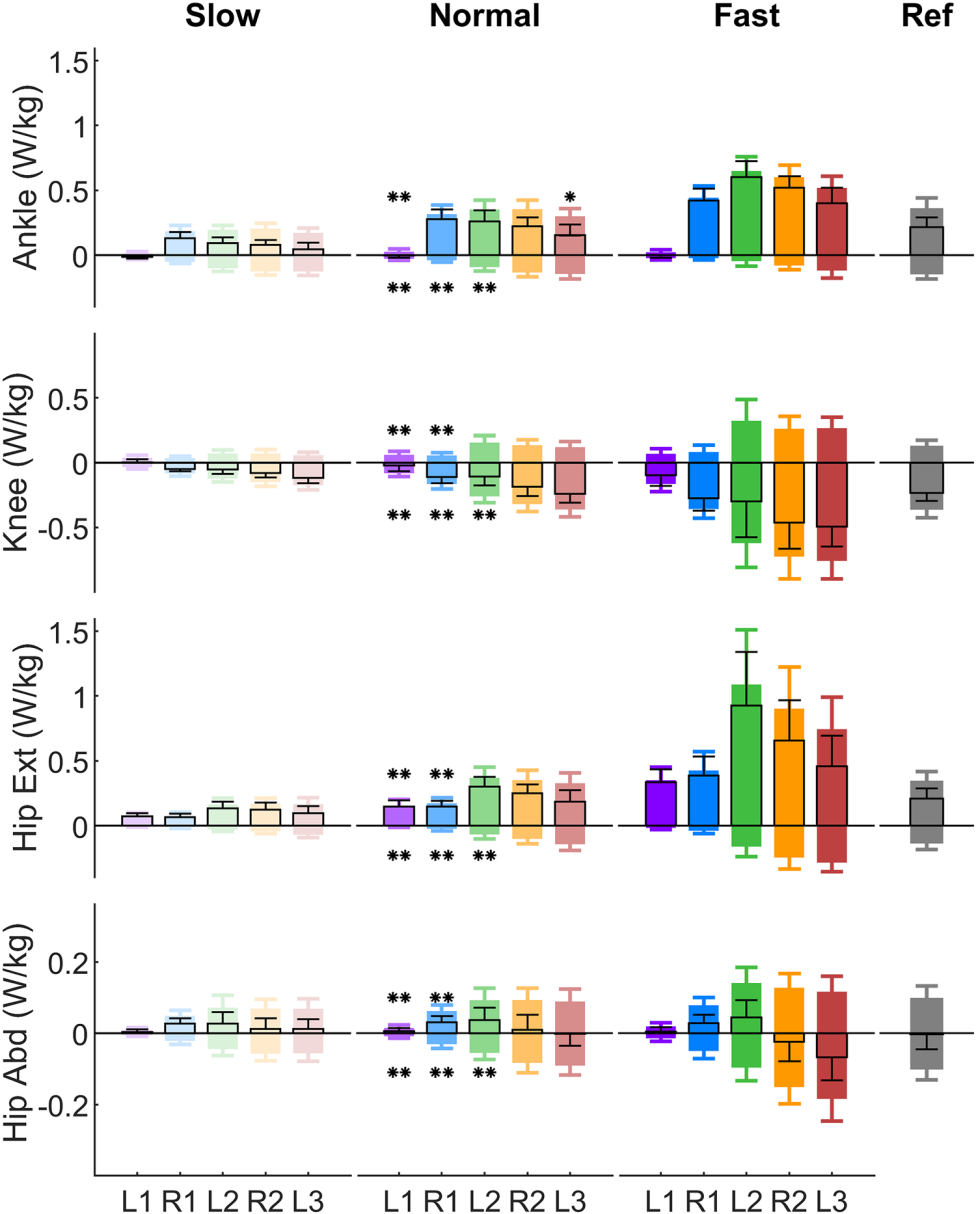
Average positive, negative and net joint power for the first five strides (L1, R1, L2, R2, L3) of slow, normal and fast target velocity gait initiation and for the reference walking (Ref). Error bars indicate $\pm \,1$ standard deviation. Positive bars indicate positive power. Negative bars indicate negative power. Black frames indicate average net joint power. The single asterisk (*) and double asterisks (**) indicate a significant difference at $p<0.05$ and $p<0.01$ with respect to Ref, respectively. Detailed statistical analysis results can be found in Supplementary Table S2.

During normal target velocity gait initiation, the ankle average positive joint power reaches the reference gait level in R1, while the negative joint power in L1, R1 and L2 are significantly smaller than the reference walking stride (Fig. 5, Supplementary Table S2). The knee negative joint power gradually increases from L1 to R2 and it reaches the reference walking stride level at R2. The knee average positive power in L1 and R1 are significantly smaller compared to the reference. The hip flexion/extension average positive joint power in both L1 and R1 are less compared to L2, R2, and L3 (Fig. 5). The knee average net power from L1 to L3 are all negative, while the average net power of the ankle and hip flexion/extension joint are positive.

Comparing the different target velocity conditions, the relative contributions of the joint average positive power are similar between the slow and the normal condition for all five strides (Fig. 4). The hip flexion/extension joint has a greater contribution in the fast condition compared to the normal condition in L1 and R1. The total average positive joint power increases with higher target velocity (Fig. 5).

#### CoM potential and kinetic energy

**Figure 6:**
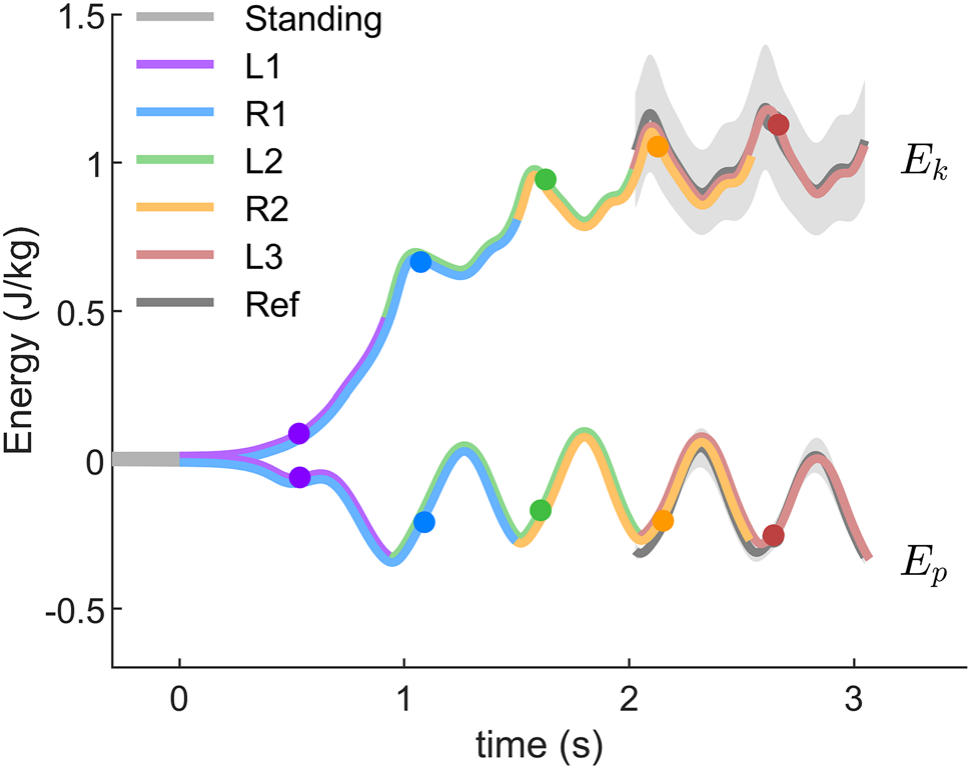
The CoM kinetic ($E_k$) and potential ($E_p$) energy during normal velocity gait initiation. Solid lines denote the mean value over 23 subjects. Time 0 is defined as the starting of the gait initiation. $E_p$ during standing is defined as zero. Ref denotes overground walking at self-selected preferred velocity. The error bands denote $\pm 1$ standard deviation. Different colors denote different strides. L1, L2, L3 denote the first, second, and third stride on the left side, respectively. R1 and R2 denote the first and second stride on the right side, respectively. The dots denote the take-off timing.

A comparison of the CoM potential and the CoM kinetic energy of gait initiation (normal target velocity) is shown in Fig. 6. For standing, the CoM potential energy was defined as zero. At the instance of the L1 TO, the amount of energy increased in the CoM kinetic energy ($0.077\pm 0.027$ J/kg) is similar to the amount of energy decreased in the CoM potential energy ($0.072\pm 0.038$ J/kg). At the beginning of L2, the CoM potential energy reaches the minimum ($-0.335\pm 0.075$ J/kg).

#### CoM mechanical power

**Figure 7:**
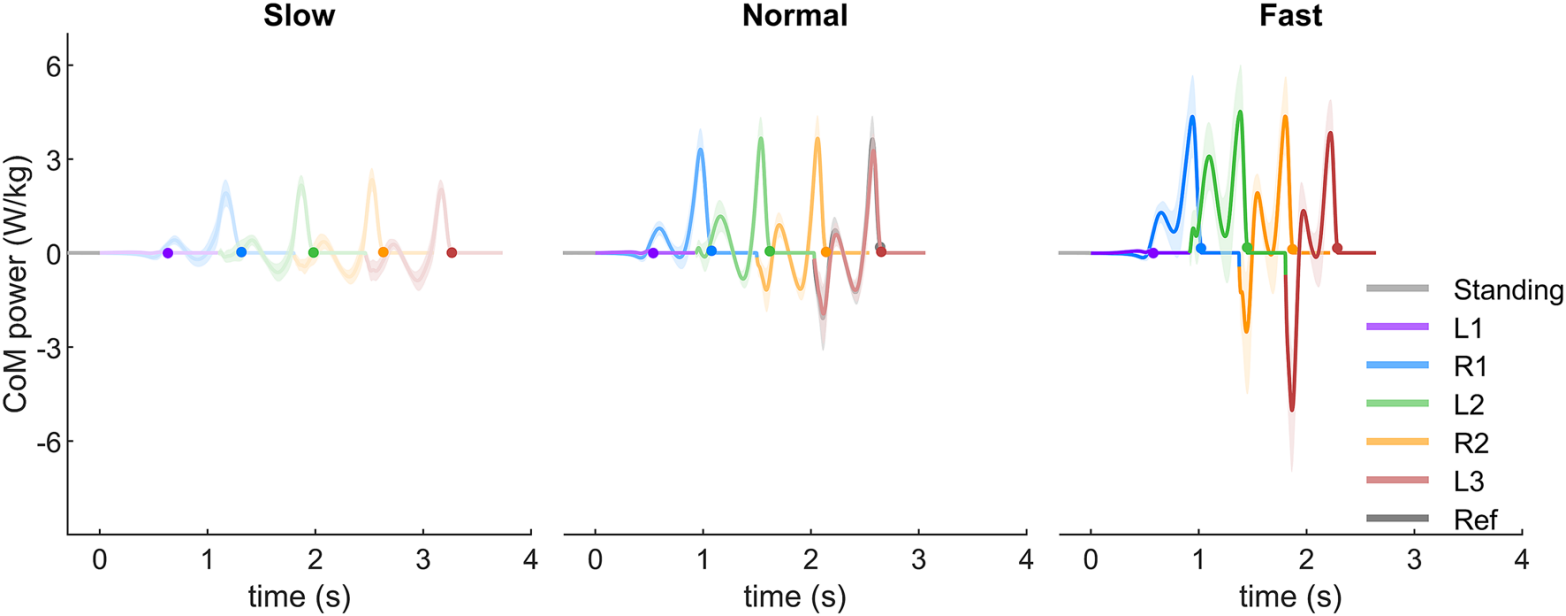
The CoM mechanical power of slow, normal and fast gait initiation. Solid lines denote the mean value over 23 subjects. Time 0 is defined as the beginning of the gait initiation. Ref denotes the regular overground walking at self-selected velocity. The error bands denote $\pm 1$ standard deviation. Different colors denote different strides. L1, L2, L3 denote the first, second, and third stride on the left side, respectively. R1 and R2 denote the first and second stride on the right side, respectively. The dots denote the take-off timing.

**Figure 8:**
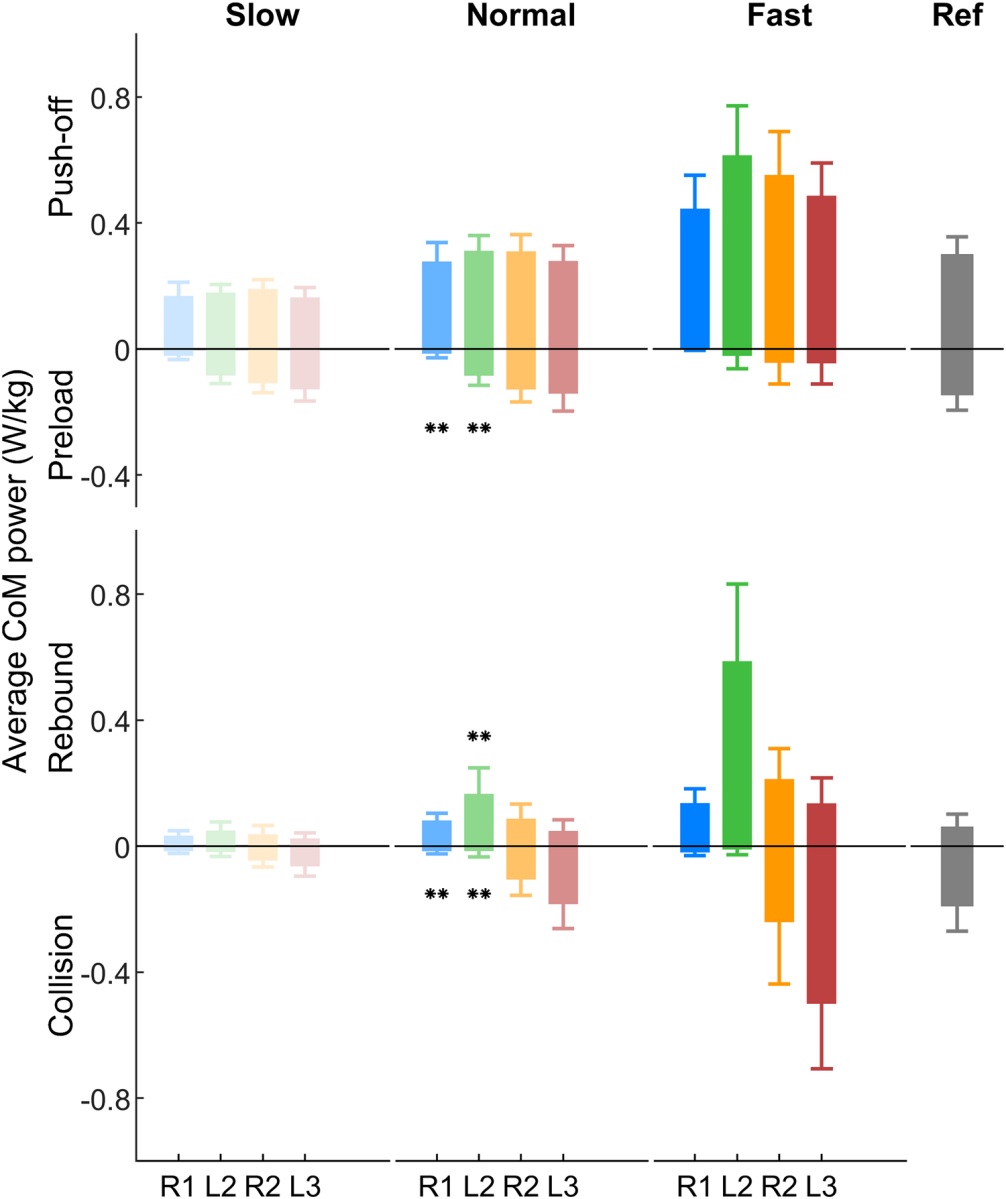
The average CoM power for slow, normal and fast gait initiation in the collision, rebound, preload and push-off phases. Error bars indicate standard deviation. L2 and L3 denote the second and third stride on the left side, respectively. R1 and R2 denote the first and second stride on the right side, respectively. Due to the low magnitude, the phase specific CoM mechanical power during L1 (the first left side stride) is not presented. The single asterisk (*) and double asterisks (**) indicate a significant difference at $p<0.05$ and $p<0.01$ with respect to Ref, respectively. Detailed statistical analysis results can be found in Supplementary Table S3.

The CoM mechanical power remains almost at zero during the first stride (L1) for all three target velocity conditions (Fig. 7). For the following strides, the CoM mechanical power shows a similar pattern as in regular walking. For the normal condition, the peak CoM mechanical power in the second stride (R1) reaches a similar magnitude as the reference stride. The CoM collision power is minimal for R1 and L2 (Figs. 7, 8), and it increases to values comparable to the reference stride within R2 and L3. The CoM rebound power in L2 is greater than all other strides for both normal and fast conditions. For the normal condition, the CoM preload power increases with an increasing number of strides, whereas the CoM push-off power remains unchanged compared to the reference. Due to the low magnitude, the phase specific CoM mechanical power during L1 was not analyzed.

## Discussion

The goal of this paper is to further understand the gait initiation mechanism and the energy sources that contribute to increase the CoM forward velocity based on a lower-limb joint-level perspective. The first five strides of gait initiation for three target velocities were analyzed in this study. Inverse kinematics and inverse dynamics analysis were used to investigate the contribution of individual joints to gait initiation. The CoM mechanical power, kinetic and potential energy analysis were used to explain joint behaviors. The results reveal the lower-limb joint functions in the frontal and sagittal planes, and provide unique insights on both the balancing and energy injection mechanism during human gait initiation.

### Joint positive power distributions

We aimed to investigate if there are specific joints, which primarily inject energy to accelerate the body during gait initiation. When reaching steady walking velocity, our data are in line with previous studies^13,19^. For steady walking (stride L3) in slow, normal and fast target velocity conditions, the average positive power at all lower-limb joints increases with walking velocity (Fig. 5). In contrast, the relative contributions of each joint to the total positive power are similar with different velocities in L3 (Fig. 4).

However, during acceleration the contributions of the hip flexion/extension joint and ankle joint are different in the first two strides (L1 and R1) of the gait initiation compared to steady state walking (Fig. 4). In L1, the hip flexion/extension joint contributes the greatest average positive power compared to the other three joints (Fig. 4). A large hip flexion peak torque can be observed after TO of the leading limb in L1 (Fig. 3). This supports our hypothesis that, in order to swing the leg forward, the hip in the leading limb contributes more during the first stride compared to the steady state (Fig. 4). In addition, the hip flexion torque created by the hip flexors can also lift the leg upwards and ensure foot ground clearance during the swing phase. Such increases in proximal muscle power output can also be observed during other dynamic movements such as human walking acceleration^20^, human sprinting^21^, and running acceleration of turkeys^22^. The ankle joint torque and power in L1 do not have the push-off pattern as typically observed in the steady state walking^23^ (Fig. 3). This is because the ankle Achilles tendon and adjacent muscles (e.g. soleus and gastrocnemius muscles) are not loaded and preactivated as in the steady state walking stride^24–26^. Therefore the ankle joint shows a lower contribution in L1 compared to steady state (Fig. 4).

Although the CoM accelerates in the first (L1) and the second (R1) strides (Fig. 2), the joint positive power contributions are different. In R1, the average positive power contribution of the ankle joint is greater than in the hip flexion/extension joint for slow and normal gait initiation (Fig. 4). Such an increase in the relative ankle contribution cannot be found during the acceleration phase of human walking or running turkeys, or during human sprinting^20–22^. This behavior can only occur for gait initiation as R1 lacks the positive hip extension power, which is typically used in continuous locomotion to accelerate the body forward.

### Strategies for accelerating the CoM forward

Based on the results of this work, we can observe four mechanisms that contribute to the forward acceleration of the CoM during gait initiation. These are (a) the transfer of potential energy to kinetic energy, (b) the excessive hip flexion of the leading limb, (c) a strong ankle push-off by the trailing limb, and (d) a minimization of collision power.

Increased lower-limb joint peak torque and power can be observed during accelerated walking^20,27^. On the contrary, our results show that the joint peak torque and power during gait initiation are not greater than steady state walking. This is because, in order to minimize the metabolic cost, humans utilize the potential energy that is present during standing to accelerate the CoM during gait initiation (Fig. 6). Our analyses on the ankle joint torque show that the CoM fore-aft velocity is created by decreasing ankle extension (plantarflexion) torque, which allows the body to fall forward like an inverted pendulum (Fig. 3). This agrees with the findings of decreased soleus muscle activity and increased tibialis muscle activity at the beginning of gait initiation^6^. It also supports the prior work using an inverted pendulum model to interpret gait initiation^5,9,28,29^. In addition, the knee joint flexes (the leg is yielding) before the TO of the leading limb (L1, Fig. 3). It indicates that the ankle joint in L1 cannot be the primary push-engine for accelerating the CoM forward. This also agrees with the theory of ballistic synergy for human normal walking^30^. After the TO of the leading limb (L1), lower-limb joints begin to inject energy to the body and accelerate the CoM forward. For instance, the leading limb hip exerts an excessive flexion torque to accelerate the leg forward. The trailing limb ankle joint shows a push-off pattern, which is similar to the push-off pattern during the steady state walking in both shape and magnitude, to redirect the CoM velocity direction and further accelerate the CoM forward (Fig. 2, 3, 6). These findings confirm our hypothesis that the potential energy has the greatest contribution to the CoM kinetic energy for the first stride, whereas for the following strides the positive joint work dominates the energy contribution. Aside from exerting positive joint power, minimizing negative joint power can also help to accelerate the CoM velocity and lower the metabolic cost. Our results indicate that this strategy is utilized by humans. For instance, all joint average negative powers in the first three strides are smaller than the steady state condition (Fig. 5). In addition, the CoM collision powers in the second and third strides are almost zero (Fig. 8).

### Balancing at the beginning of gait initiation

In order to maintain balance, the CoM has to be shifted towards the trailing limb (stance leg) before the lifting of the leading limb (swing leg) (Fig. 1). This requires greater leg force in the leading limb compared to the trailing limb (Fig. 2). This behavior was shown in prior work, which also demonstrated the lateral shift of the CoP at the beginning of gait initiation^2,10,31^. Jian et al.^10^ speculated that the CoP movement is due to a momentary loading of the swing limb and an unloading of the stance limb. None of these studies addressed how the weight shift is achieved on the joint level.

Our results demonstrate that the trailing limb force decreases and the leading limb force increases (weight shifting initiation phase, P1, Fig. 1), while both patterns are similar (inverted). This indicates that humans prefer to keep the CoM vertical acceleration zero while shifting the CoM laterally. This could be done by extending the leading limb (e.g. ankle plantarflexion) and shortening the trailing limb (e.g. hip and knee flexion), which potentially requires complex control for the coordination of the muscles of both legs. Another way to achieve this is to exert abduction torque on the hip joint of the leading limb or adduction torque on the hip joint of the trailing limb. This is a simpler approach since it only requires the control of one joint. The increase in hip abduction torque (Fig. 3) supports our hypothesis that humans use hip abduction torque of the leading limb to realize the initial lateral weight shift.

### The emergence of the walking pattern

The vertical GRF during the stance phase of walking is characteristically M-shaped^32,33^, which is attributed to leg compliance^34^. Surprisingly, the trailing limb exhibits the M-shape pattern after the weight shifting initiation phase (P1, Fig. 1). Furthermore, the pattern of the CoM mechanical power in R1 also shows similar rebound and push-off behaviors as in normal walking (Figs. 7, 8). These findings indicate that the compliant leg behavior for walking has already emerged after TO of the leading limb.

Our results indicate there could be a time order of switching the joint control from standing to walking to manage gait initiation for both lower limbs. Such an order could be interpreted based on the similarity of joint torque patterns during gait initiation to the patterns during steady state walking. We found that first the leading limb hip flexion/extension joint shows similar torque at TO of L1, both in shape and magnitude, as the steady state walking stride (Fig. 3). Following, the steady state-like torque pattern in the trailing limb ankle, knee and hip abduction/adduction joint can be observed. After the touch-down (TD) of L1, all joints show similar patterns (angle, torque, power) as in steady state walking. The similar behaviors can also be found in the slow and the fast gait initiation condition (see Supplementary Fig. S1, S2, and S3).

### Future work

It has been shown that there are gender differences in the lower-limb joint kinematics during walking^35,36^. For future work, we will extend the experiments with more female and male subjects and investigate if the gender differences found in walking can also be observed during gait initiation. This work focused on the biomechanical analysis of human gait initiation from the lower-limb joint perspective. In future studies, we plan to develop simplified and multi-segment musculoskeletal walking models that are capable of reproducing the gait initiation features observed in this work. Simplified models, such as the conceptual models from^37,38^ could potentially be extended to 3D and to reproduce the hip joint function for gait initiation. The multi-segment musculoskeletal walking models (e.g. models from^39,40^) could benefit from an optimization of the control parameters (e.g. feedback gains) based on the human gait initiation data. In future experiments, we plan to measure human lower-limb and trunk muscle activations which could be used to verify the musculoskeletal models. Such simplified and multi-segment complex model could provide further insights on how humans generate different gaits at mechanical and neuromuscular level. Furthermore, the models could also be used as part of a controller for prostheses and exoskeletons (e.g. studies from^41–43^) to support human gait of those who need assistance^44^.

## Conclusion

This work aims at further understanding gait initiation mechanism by focusing on the energy injection from the perspective of the lower-limb joints. This study provides novel insights on the coupling between the frontal and sagittal plane joints during gait initiation. We found that the hip abduction torque on the leading limb (lifting leg) is the primary driver of the lateral weight shift at the beginning of gait initiation. The walking gait pattern in both overall leg behavior and lower-limb joint behavior emerges after the TO of the leading limb. The hip flexion/extension joint has the greatest contribution to the joint positive power in the first stride while the ankle joint contributes the most during the second stride. The peak torque and power of all joints during gait initiation are not greater than the steady state walking condition.

The results of this study provide a gait initiation dataset of young and non-mobility impaired humans. The dataset could be used by therapists or clinicians to understand the fundamentals of gait initiation including balance and body propulsion. The dataset could also be used as a reference for the diagnosis of gait impairments and the identification of possible sources of gait disorders. With that knowledge, therapists or clinicians could find the strategies for the physical therapy or other kinds of medical treatments. Additionally, joint kinematics and kinetics can be used to develop algorithms for recognizing human intention for gait initiation. The data and our outcomes can potentially help to develop control principles and techniques to improve wearable assistive devices such as exoskeletons and prostheses. In addition, bipedal robot control could be inspired by the presented human gait initiation strategies (e.g. the weight shifting mechanism).

## Methods

### Subjects

Twenty three young healthy subjects (11 females, 12 males, age $25.2\pm 3.8$ years, body mass $67.0\pm 14.3$ kg, height $1.74\pm 0.12$ m, mean ± std) were enrolled in this study. All subjects were healthy without any neuromuscular injury or functional impairment. This study was approved by the Ethics Committee of TU Darmstadt and was carried out based on the guidelines of the Declaration of Helsinki. All subjects gave written informed consent.

### Experimental setup

**Figure 9:**
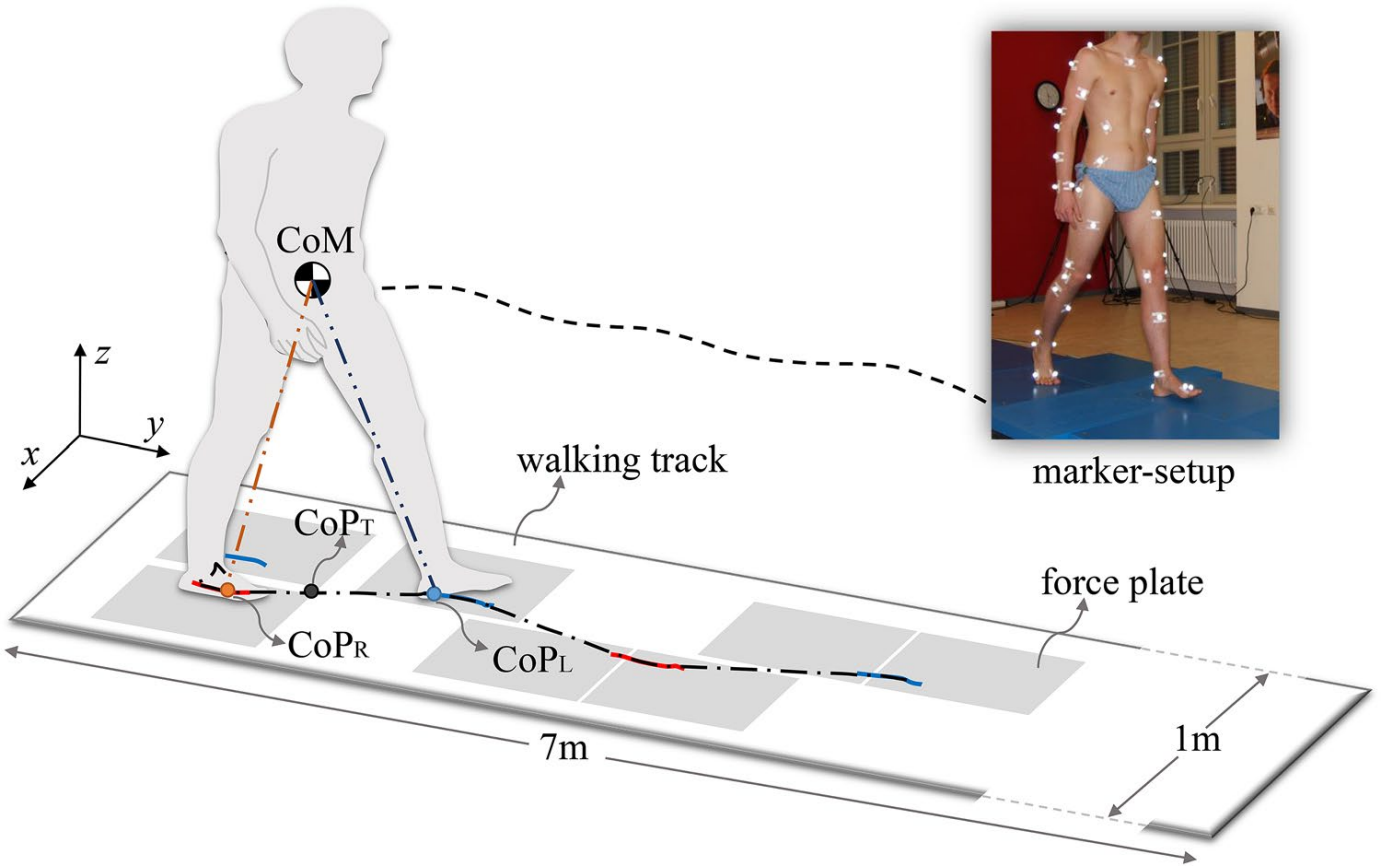
Experimental setup overview. $\mathrm {CoP_L}$, $\mathrm {CoP_R}$ and $\mathrm {CoP_T}$ denote the left leg, right leg and total center of pressure, respectively.

A 7 m long, 1 m wide flat walking track was constructed for this experiment (Fig. 9). Seven force plates (five 9260AA and two 9287C, Kistler, Switzerland) were firmly mounted on a metal frame and embedded in the track. Ground reaction forces (GRF) were recorded at 1 kHz. The positions of the force plates were carefully arranged so that they could measure the GRFs of each leg during standing and the first three strides for the gait initiation trails. A 3D motion capture system (ten high-speed cameras, model Oqus, Qualisys, Sweden) recorded full body kinematics from 51 reflective markers at 500Hz.

### Experimental protocol

Prior to data collection, subjects performed several different walking trials to: (1) warm up and familiarize themselves with the setup, (2) test whether their step length was appropriate for the setup, (3) determine the appropriate starting locations so that the GRFs of the first three strides could be captured. Then, there are reference walking trails and gait initiation trials. For reference walking trials, subjects performed walking trials (8 repetitions) on the instrumented track with their preferred walking velocity. Subjects were instructed to start walking at least 3 m before he/she stepped on the first force plate. For gait initiation trials, subjects were instructed to stand as still as possible on the first two force plates. Subjects started walking at self-selected time, following an auditory cue, with three different self-selected target velocities (slow, normal and fast, 8 repetitions for each velocity). Subjects always started walking with left leg due to the force plate arrangement. All experiments were conducted barefoot.

### Data processing

The beginning of gait initiation was defined as the moment when the displacement between the CoP and the CoM in walking direction was larger than 1.5cm. The vertical GRF was used to detect the TD and the TO events. Gait initiation trial data were separated into standing, L1, R1, L2, R2 and L3 (as shown in Fig. 1). Standing was defined from the beginning of the experiment until the beginning of gait initiation. L1 and R1 were defined from the beginning of gait initiation until the first TD of the left and right leg, respectively. L2, R2 and L3 were defined as the following second left, second right, and third left strides, respectively. Each stride was defined as TD to TD of the same leg (Fig. 1).

The whole body CoM positions and velocities, and lower-limb joint kinematics and kinetics were computed with the open-source OpenSim software (version 4.1)^45^ using a full body model adapted from^46^. The CoM power for the left and the right legs were calculated as the dot product of the CoM velocity and the left and right GRFs, respectively. Joint kinetics data were normalized to the individual subject body mass. GRFs were normalized to the individual subject body weight. Joint net, positive and negative work during each stride (e.g. L1, R1, L2 etc.) were calculated by integrating the joint power over one stride period, positive power period, and negative power period, respectively. Average joint positive and negative power were calculated by the joint positive and negative work divided by the stride period, respectively^13^. The average CoM power were calculated using the individual limb method^47^. All data were processed with Matlab (R2020a, MathWorks) scripts.

### Statistics

Statistical analysis was not conducted to analyze all the data because of its large amount. Here, the statistical analyses were performed for the average joint power (both positive and negative power, Fig. 5, Supplementary Table S2) and the average CoM power (Fig. 8, Supplementary Table S3) during each stride. All data were assessed for normality using the Jarque-Bera test. If the data were normally distributed, a one-way repeated measures analysis of variance (ANOVA) was performed across the strides (i.e. L1, R1, L2, R2, L3 and the reference stride). Otherwise, the nonparametric Kruskal–Wallis test was used for differences between groups. The Mauchly test was used to evaluate sphericity. The Greenhouse–Geiser correction was applied if the sphericity assumption was violated. If the repeated measures ANOVA or the Friedman test indicated a significant effect, the paired *t* test was used for post hoc tests. The paired *t* test was performed between each individual stride during gait initiation and the steady state condition stride. A statistical difference was considered at a level of $p<0.05$. A single asterisk (*) and a double asterisks (**) indicate the significant difference of $p<0.05$ and $p<0.01$ with respect to the reference (Ref), respectively. All statistical tests were conducted in Matlab (R2020a, MathWorks).

## Supplementary Information

Supplementary Information.

## Data Availability

Additional data and figures are available in the Supplementary Information.
