# Supplementary material for: The mechanisms and mechanical energy of human gait initiation from the lower-limb joint level perspective
Source: Sci Rep. 2021 Nov 18;11:22473. doi: 10.1038/s41598-021-01694-5 (PMC8602421; doi:10.1038/s41598-021-01694-5)
Supplement: Supplementary file 1 — Supplementary Information. [file 41598_2021_1694_MOESM1_ESM.pdf]

Supplementary material to:

The mechanisms and mechanical energy of human gait initiation from the lower-limb joint level perspective

Guoping Zhao[1,*], Martin Grimmer[1], and Andre Seyfarth[1]

[1]Lauflabor Locomotion Laboratory, Centre for Cognitive Science, Technical University of Darmstadt, 64289 Darmstadt, Germany
[*]Corresponding author, guoping.zhao@tu-darmstadt.de

Mean CoM velocity

The mean CoM velocity for five consecutive strides (i.e. L1, R1, L2, R2, and L3) during gait initiation with three different target velocities are shown in Table S1.

Table S 1. Mean center of mass (CoM) velocity for five consecutive strides during gait initiation with three different target velocities. Mean±1standard deviation. The unit is m/s.

Target velocity	L1	R1	L2	R2	L3
slow	0.24 ± 0.04	0.45 ± 0.05	0.84 ± 0.07	0.93 ± 0.07	0.97 ± 0.08
normal	0.35 ± 0.04	0.65 ± 0.05	1.21 ± 0.10	1.33 ± 0.10	1.38 ± 0.10
fast	0.47 ± 0.05	0.85 ± 0.08	1.75 ± 0.17	1.94 ± 0.15	1.99 ± 0.13

Comparison of joint average power

The statistical analysis results of the average joint power (both positive and negative power) for normal gait initiation during each stride are shown in Table S2.

Comparison of CoM average power

The statistical analysis results of the average CoM power for normal gait initiation in the collision, rebound, preload and push-off phases during each strides are shown in Table S3.

Joint kinematics and kinetics during slow and fast gait initiation

The lower-limb joint kinematics and kinetics during slow, normal and fast target velocity are shown in Fig. S1 , Fig. S2 and Fig. S3, respectively.

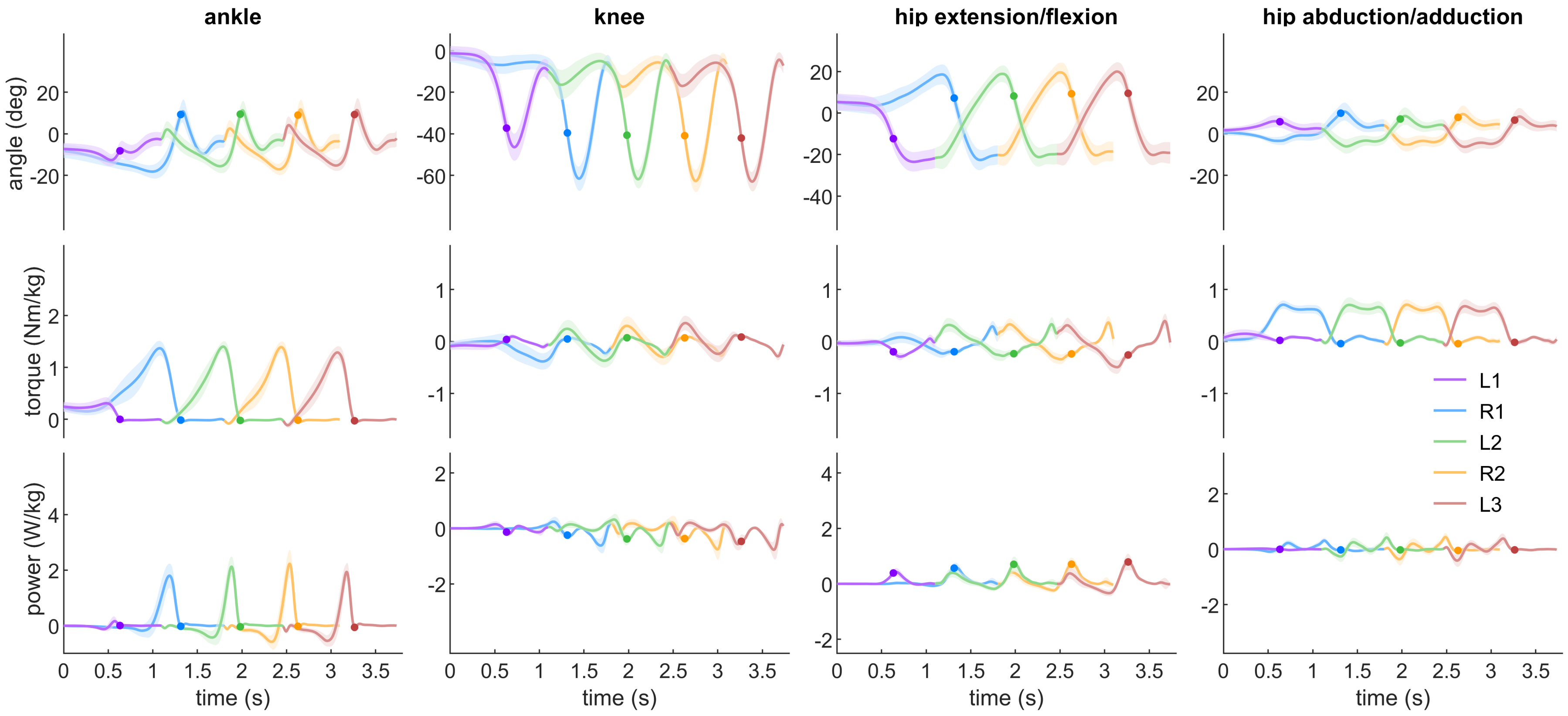

Figure S 1. Ankle extension/flexion, knee extension/flexion, hip extension/flexion and hip abduction/adduction joint angle, torque and power during the slow target velocity gait initiation. Extension and abduction angle, torque and power are defined as positive. Solid lines denote the mean value over 23 subjects. The error bands denote ±1 standard deviation. Time 0 is defined as the starting of the gait initiation. Different colors denote different strides. L1, L2, L3 denote the first, second, and third stride on the left side, respectively. R1 and R2 denote the first and second stride on the right side. Ref denotes the regular overground walking at self-selected preferred velocity. The dots denote the take-off moment.

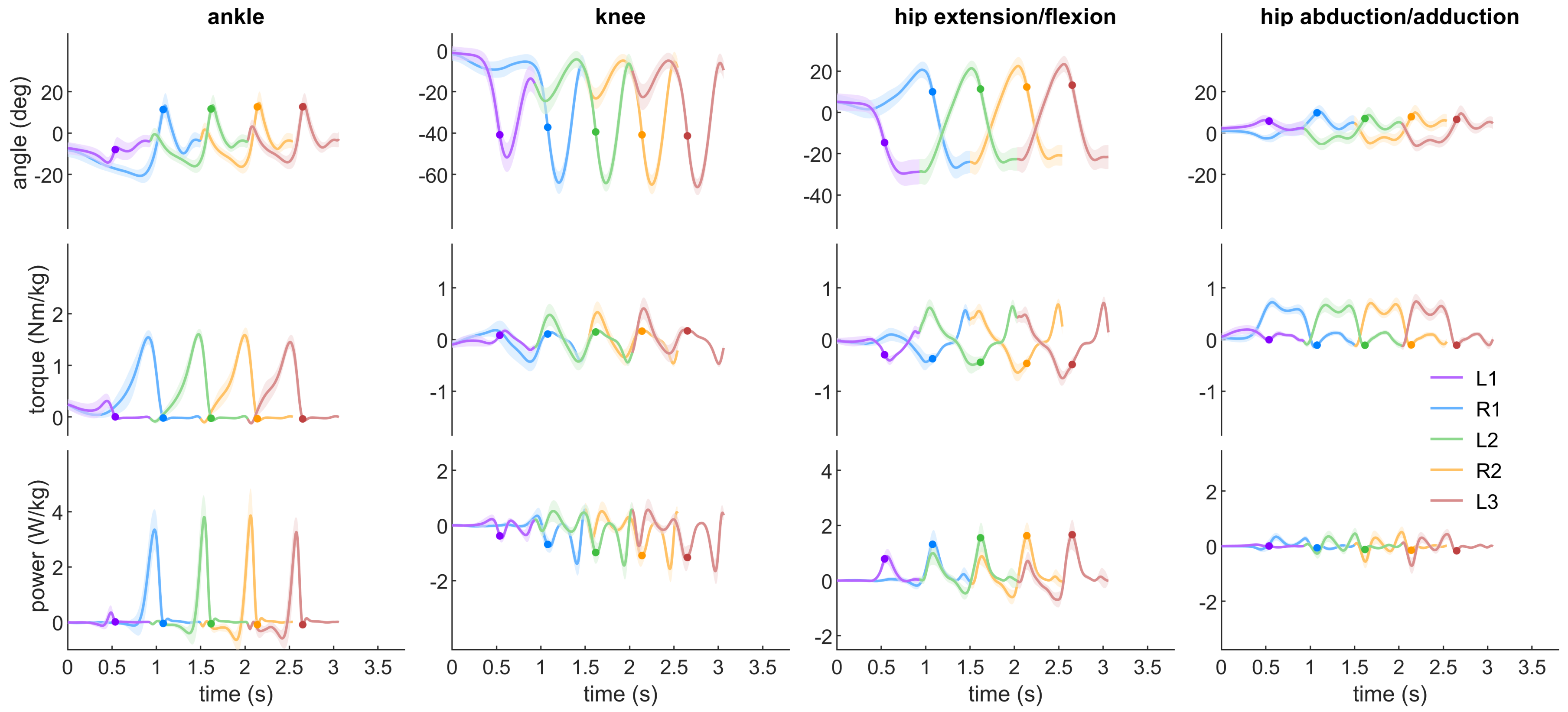

Figure S 2. Ankle extension/flexion, knee extension/flexion, hip extension/flexion and hip abduction/adduction angle, torque and power during the normal velocity gait initiation. Hip and knee extension, ankle plantarflexion, hip abduction, and extension torque are defined as positive. Solid lines denote the mean value over 23 subjects. The error bands denote ±1 standard deviation. Time 0 is defined as the beginning of the gait initiation. Different colors denote different strides. L1, L2, L3 denote the first, second, and third stride on the left side, respectively. R1 and R2 denote the first and second stride on the right side. Ref denotes the regular overground walking at self-selected preferred velocity. The dots denote the take-off timing.

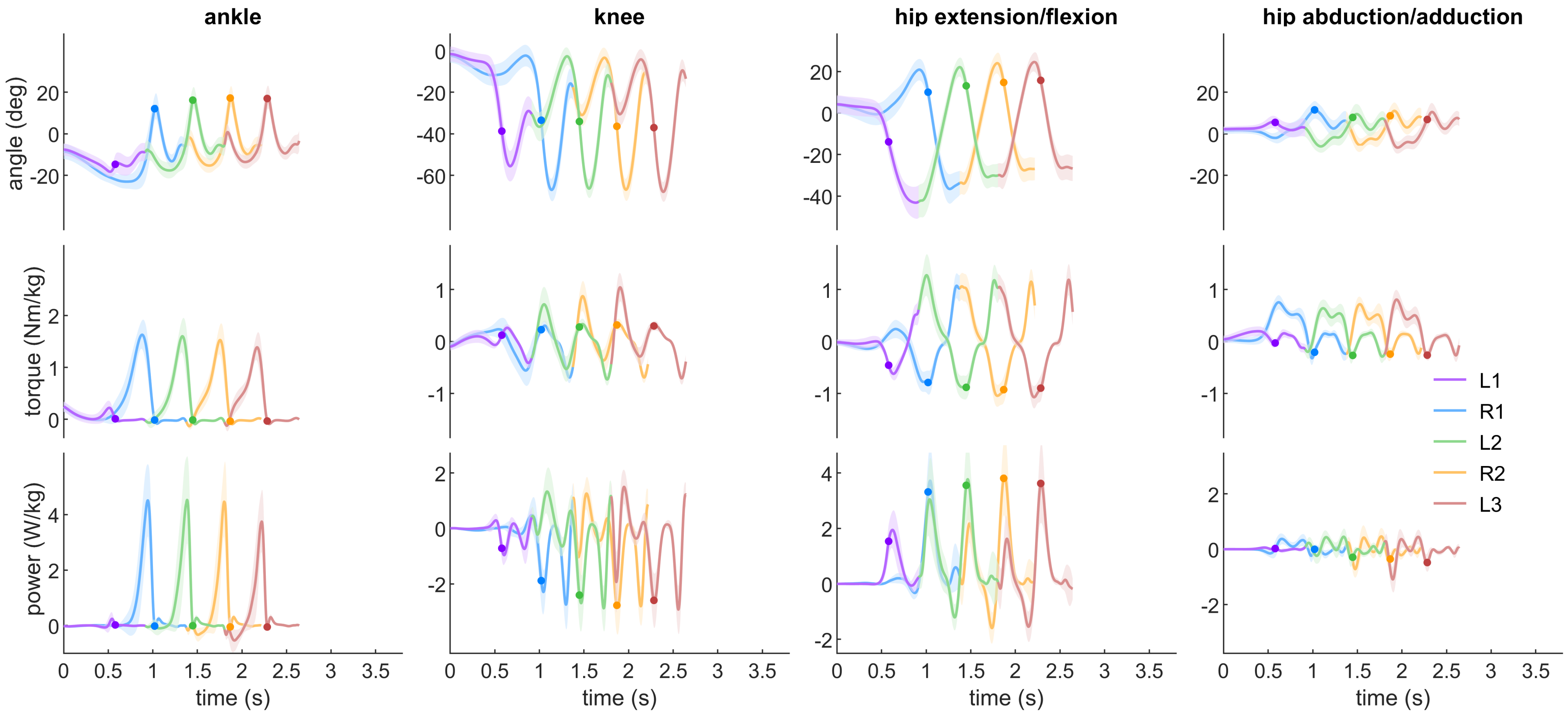

Figure S 3. Ankle extension/flexion, knee extension/flexion, hip extension/flexion and hip abduction/adduction joint angle, torque and power during the fast target velocity gait initiation. Extension and abduction angle, torque and power are defined as positive. Solid lines denote the mean value over 23 subjects. The error bands denote ±1 standard deviation. Time 0 is defined as the starting of the gait initiation. Different colors denote different strides. L1, L2, L3 denote the first, second, and third stride on the left side, respectively. R1 and R2 denote the first and second stride on the right side. Ref denotes the regular overground walking at self-selected preferred velocity. The dots denote the take-off moment.

Table S 2. Joint average power (positive and negative) comparison between the five gait initiation strides (i.e. L1, R1, L2, R2, and L3) and the reference stride. The repeated measures ANOVA results ($F() =, p$) or the Kruskal-Wallis results ($\chi^2_K() =, p$) are reported for each joint. p-values of the *post hoc* test results are reported for each joint during every stride.

Joint			L1	R1	L2	R2	L3
Ankle	positive	$F(5,110) = 176.4$, $p < 0.001$	< **0.001**	0.227	1.000	1.000	**0.020**
	negative	$F(5,110) = 142.8$, $p < 0.001$	< **0.001**	< **0.001**	< **0.001**	0.827	1.000
Knee	positive	$\chi^2_K(5) = 74.2$, $p < 0.001$	< **0.001**	< **0.001**	0.962	1.000	1.000
	negative	$\chi^2_K(5) = 108.8$, $p < 0.001$	< **0.001**	< **0.001**	< **0.006**	0.871	1.000
Hip fle/ext	positive	$F(5,110) = 116.6$, $p < 0.001$	< **0.001**	< **0.001**	0.994	1.000	0.998
	negative	$\chi^2_K(5) = 104.6$, $p < 0.001$	< **0.001**	< **0.001**	< **0.009**	0.832	1.000
Hip abd/add	positive	$\chi^2_K(5) = 71.8$, $p < 0.001$	< **0.001**	**0.008**	1.000	1.000	0.998
	negative	$F(5,110) = 151.3$, $p < 0.001$	< **0.001**	< **0.001**	< **0.001**	0.064	0.770

Table S 3. The CoM average power comparison between the four gait initiation strides (i.e. R1, L2, R2, and L3) and the reference stride. The repeated measures ANOVA results ($F() =, p$) are reported for each phase. p-values of the *post hoc* test results are reported for each phase during every stride.

Phase		R1	L2	R2	L3
Push-off	$F(4,88) = 5.2$, $p = 0.008$	0.765	0.999	1.000	0.843
Preload	$F(4,88) = 110.8$, $p < 0.001$	< **0.001**	< **0.001**	0.728	1.000
Rebound	$\chi^2_K(4) = 50.4$, $p < 0.001$	0.270	< **0.001**	0.348	0.992
Collision	$\chi^2_K(4) = 88.9$, $p < 0.001$	< **0.001**	< **0.001**	0.105	1.000
